# Using saliva epigenetic data to develop and validate a multivariable predictor of esophageal cancer status

**DOI:** 10.2217/epi-2023-0248

**Published:** 2024-01-16

**Authors:** Timothy C Stone, Vanessa Ward, Aine Hogan, Kai Man Alexander Ho, Ash Wilson, Hazel McBain, Margaret Duku, Paul Wolfson, Sharon Cheung, Avi Rosenfeld, Laurence B Lovat

**Affiliations:** 1 Division of Surgery & Interventional Science, University College London, Charles Bell House, 43-45 Foley Street, London, W1W 7TY, UK; 2 Wellcome/EPSRC Centre for Interventional & Surgical Sciences (WEISS), University College London, Charles Bell House, 43-45 Foley Street, London, W1W 7TY, UK; 3 Department of Gastrointestinal Services, University College London Hospital, 235 Euston Road, London, NW1 2BU, UK; 4 Department of Computer Science, Jerusalem College of Technology, Havaad Haleumi 21, Givat Mordechai, 91160, Jerusalem, Israel

**Keywords:** biomarker panel, diagnosis, epigenetics, esophageal adenocarcinoma, saliva

## Abstract

**Background:**

Salivary epigenetic biomarkers may detect esophageal cancer.

**Methods:**

A total of 256 saliva samples from esophageal adenocarcinoma patients and matched volunteers were analyzed with Illumina EPIC methylation arrays. Three datasets were created, using 64% for discovery, 16% for testing and 20% for validation. Modules of gene-based methylation probes were created using weighted gene coexpression network analysis. Module significance to disease and gene importance to module were determined and a random forest classifier generated using best-scoring gene-related epigenetic probes. A cost-sensitive wrapper algorithm maximized cancer diagnosis.

**Results:**

Using age, sex and seven probes, esophageal adenocarcinoma was detected with area under the curve of 0.72 in discovery, 0.73 in testing and 0.75 in validation datasets. Cancer sensitivity was 88% with specificity of 31%.

**Conclusion:**

We have demonstrated a potentially clinically viable classifier of esophageal cancer based on saliva methylation.

Esophageal cancer has one of the lowest 5-year survival rates (20%) of all cancers that were diagnosed in the USA from 2010 to 2016 [1]. This contrasts starkly with rates for prostate cancer (98%), melanoma of the skin (92%) or breast cancer (90%). Similar rates for this disease have been observed worldwide. The disease is the sixth leading cause of mortality related to cancer and the eighth most common cancer globally [2]. There are two histological subtypes: squamous cell carcinoma and adenocarcinoma (AdCa). Whereas squamous cell carcinoma is the commonest subtype in the global south, AdCa is the predominant type in the global north, and the UK is the world epicenter for this disease [3]. Esophageal AdCa develops almost entirely in patients with the premalignant condition Barrett’s esophagus, which is known to progress through low- then high-grade dysplasia (HGD) to intramucosal AdCa before it finally becomes invasive [4]. While it is easily and reliably treated endoscopically at all stages including intramucosal AdCa, as soon as it becomes invasive disease, the prognosis rapidly worsens [5–7]. Early detection is therefore key.

Clinical symptoms of esophageal cancer are less likely to come to the attention of individuals who have the disease until it has progressed to an advanced stage [8,9]. The disease may only present itself with clinically relevant symptoms at the point where the tumor has reached a point of extensive infiltration [10]. Diagnostic tests for the early identification of the disease could therefore have a considerable impact on favorable prognostic outcomes.

Salivary diagnostics is well established for the detection of certain conditions (oral diseases [11], autoimmune conditions [12], HIV [13]). Epigenetic-based salivary biomarkers hold great promise but have currently not yet been implemented in a clinical setting. They would be very easy to use. A saliva sample can be provided by the patient in their own home. Such a convenient, self-administered procedure would eliminate the need for the patient to visit a clinic, freeing the valuable time of healthcare staff and reducing social exposure risks for everyone. Saliva has many advantages over blood. It does not clot and is easier to store [14]. Saliva is stable in preservative and maintains viable DNA at room temperature for at least 8 months [1,15] while inactivating viral particles. Furthermore (and in spite of the recent pandemic), saliva remains a safer material than blood to store, ship and work with.

All cancers can induce an extensive range of epigenetic changes, with both hypomethylation [16] and hypermethylation [2,17] widely prevalent. These changes can often precede more severe clinical phenotypes [18]. Detection of methylation changes in the body therefore has the potential to act as a valuable marker for early diagnosis. Furthermore, these epigenetic changes can be detected beyond the tissue of tumor origin. Cancer biomarkers based on DNA methylation outside the tissue of origin have been proposed using blood [19,20], but multiple studies also suggest that saliva could have similar potential. For example, a meta-analysis of 18 salivary DNA methylation panel studies has shown its value to diagnose new head and neck cancers [21]. Changes in salivary DNA methylation can also detect cancer recurrence [22]. Beyond this, DNA hypermethylation in sputum is a promising tool for early detection of lung cancer [23]. It is not yet known whether saliva can be used as a detection tool for esophageal cancer. The aim of this study was to develop and validate a multivariable predictor of esophageal AdCa (but not squamous cell carcinoma) status using salivary epigenetics.

## Materials & methods

### Study design & participant recruitment

The study design is an observational case–control study. Participants were obtained via voluntary recruitment. Ethical approval for the study was obtained (see ‘Ethical conduct of research’). All patients undergoing secondary care assessment of suspected esophageal cancer or surveillance endoscopy for the preneoplastic lesion Barrett’s esophagus and who had never had any cancer-related treatment were eligible for the study. Exclusions included patients unfit for endoscopy due to comorbidity, or for endoscopic biopsy due to bleeding disorders. They were recruited at 19 hospital sites across the UK between October 2018 and July 2021. In addition, we recruited healthy volunteers (HVs), who included family members or friends who were attending the hospital with patients; additionally, some HV people were also recruited outside of the hospital environment. No HV had any previous history of any cancer. All patients and volunteers signed a written consent form after receiving a detailed information sheet that had been approved by the Ethics Committee.

**Figure 1.**
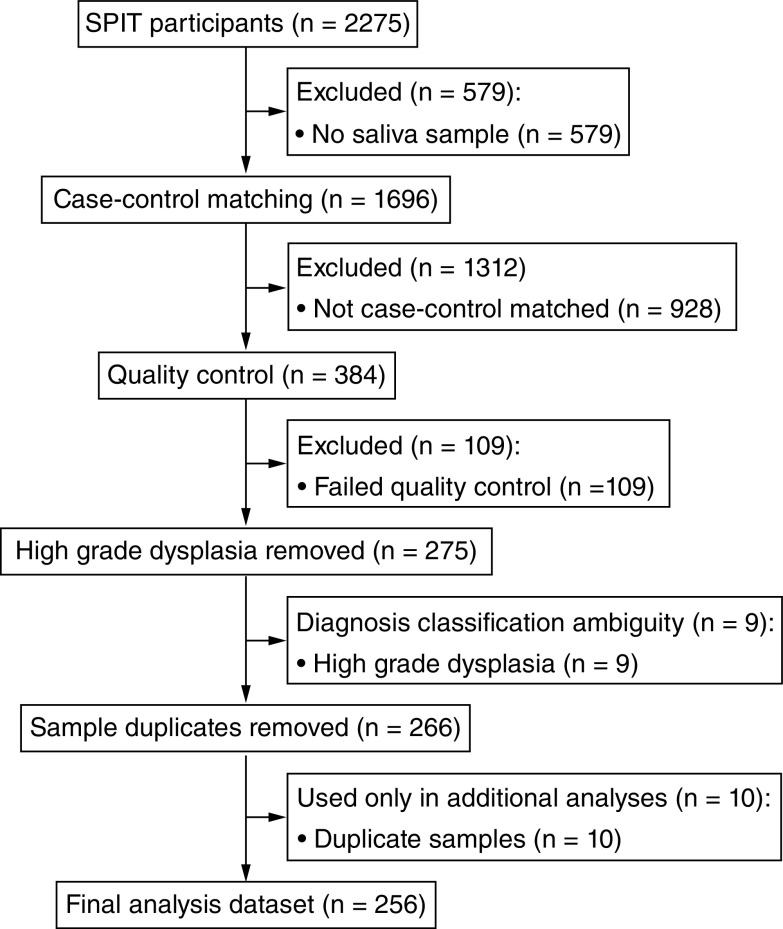
CONSORT diagram.

Figure 1 shows a Consort diagram summarizing the acquisition of participant data. A total of 2275 people were recruited to the Saliva to Predict Disease Risk (SPIT) study. Of these, only 1696 people contributed saliva samples. We selected 384 Caucasian people for inclusion into the final array analysis using case–control matching as we did not have adequate numbers of samples from non-Caucasians with cancer. A total of 109 people failed array quality control (QC), leaving 275 individuals. Of these, nine samples were from patients with HGD which were not used in the main analysis (see next section) and ten samples were technical replicates used to assess reproducibility. This left us with 256 samples for the principal analysis.

### Sample collection

Saliva samples were collected using the Oragene^®^ DNA OG/600 saliva collection kit (DNAGenotek, Ottawa, Canada). All participants were required to fast for a minimum of 1 hour prior to sample collection. Sample collection for most non-HV participants was performed at the site hospital, prior to the subject receiving an endoscopy. In the case of the HV group (who did not have any endoscopy), sample collection was done in their own time, following detailed written instructions in line with the manufacturer’s instructions [24] which explicitly stated the need to fast before sample collection for at least 1 hour. We recommended sample submission upon waking. We instructed volunteers to seal the samples immediately after donation and provided a prepaid envelope for easy postal return at room temperature. Samples were stored at -80°C upon receipt at our center. Samples were frozen within 7 days of collection, although DNA collected with this system is known to remain stable at room temperature for many months [15].

### Sample processing

DNA extraction was undertaken using the Zymo Quick-DNA™ Miniprep Plus Kit (Cambridge Bioscience, Cambridge, UK). Briefly, saliva was thawed and cells were lysed using proteinase K. DNA was then isolated by digesting the sample with genomic binding buffer before it was purified using a Zymo-Spin™ IIC-XL column in a collection tube. The sample was centrifuged and the flow-through was discarded. The DNA was then eluted using a DNA elution buffer before being centrifuged again.

DNA quantification was undertaken using the Bioanalyzer 2100 (Agilent, CA, USA), a microfluidic chip-based automated capillary electrophoresis machine. It yields highly precise analytical evaluation of DNA, RNA and protein integrity and quantity. We used the Bioanalyzer High Sensitivity Assay Kit (Agilent), following the standard manufacturer’s protocol.

A sample was acceptable for the next step only if the DNA/protein optical density ratio at 260/280 nm was above 1.8 and the contamination with protein (absorption at 230 nm) was low (260/230 ratio was above 1.5) The pellet was then frozen at -80°C until use.

### Bisulfite conversion

The Zymo EZ-96 DNA Methylation Kit (Zymo Research, CA, USA) was used. Optimal DNA input to the bisulfite conversion process is 200–500 ng/μl. The input volume was calculated by dividing the total DNA input (ng) by DNA sample concentration (ng/μl.) The bisulfite conversion process followed standard protocols [25]. Briefly, after addition of Zymo M-dilution buffer, the final volume was adjusted to 50 μl with UltraPure DNase/RNase-Free Distilled Water. The sample was incubated at 95°C for 30 s, followed by 50°C for 60 min × 16 cycles, then held at 4°C. On the following day, the samples were incubated with binding buffer and loaded into the wells of the binding plate, then repeatedly centrifuged. Flow-through was discarded. The bisulfite-converted bound sample was then eluted and stored at -80°C until used.

### Array plate experiments & duplication

**Figure 2.**
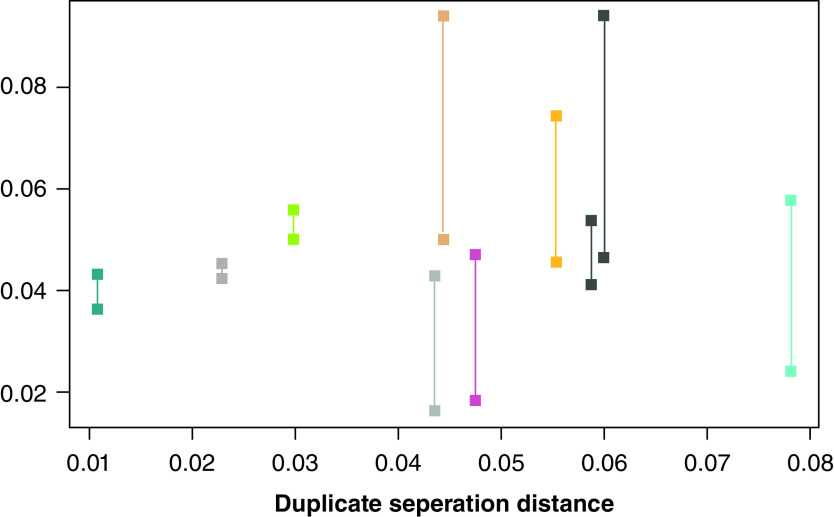
Plot of Kolmogorov–Smirnoff distance metric for biological duplicates showing distances between duplicates (x-axis) and distances from each array to a summarized average array (y-axis).

Two methylation array sets were collected, which were scanned in July 2020 and January 2022. An important question that has raised concerns about methylation array technology is how well probes replicate, especially given the delay between the two experiments. We were concerned to determine whether the two experiments could be combined. We included ten biological replicates to gain insight into this and to inform our approach to QC. We extracted the distance metric that is part of a standard Kolmogorov–Smirnoff (KS) significance test of the β-values and noted the distance of the duplicates from each other. We also created an ‘average array’ of the several hundred other samples in our experiment. We plotted the duplicate pairs on a graph (Figure 2). The x-axis is the duplicate pair distance (which is the same value for each pair because the distance from A to B is the same as the distance from B to A). The y-axis contains the distance of each array to the summarized methylation values (the average array). Only four of the ten duplicate pairs replicated well. We did, however, observe that the pairs that replicated well had an average β-KS distance of between 0.3 and 0.6 and that the worst-performing pairs contained single samples that exceeded 0.6. As a consequence of this, we applied this informal rule to choose the duplicate samples that were likely to be the more reliable pairs. We agree with the findings reported in the literature that there are replication issues with EPIC arrays [26]. Exerting highly stringent QC may lead to discarding arrays where the KS distance of the array to the mean of all array values exceeds 0.6, but this is likely to remove good samples along with the bad.

### Study size & case–control matching

Our priorities were to include as many disease phenotypes as possible and to match the controls to the disease cohort so that any potentially confounding covariates would be minimized. We had ten duplicate samples, so we had to restrict ourselves to a single one of these.

We decided to include intramucosal esophageal adenocarcinoma (IMC) and invasive esophageal AdCa in model generation, but relegated HGD subjects to independent testing only, due to diagnostic and categorical ambiguities that could be present in that latter group, which subsequent analyses appeared to vindicate.

We developed procedures for selection and assignment of the samples to the array plate. Batch effects in this technology can be large [27]. If these effects are confounded with nontechnical covariates, statistical batch correction risks removing additional information of potential biological interest. We therefore took measures to ensure that the main batch variables (the two array plates and the 12 rows of each individual plate) had as much biological and clinical homogeneity as possible at the design stage. This was done via a three-step process. In the first step, case–control matching was performed. We constructed a set of multidimensional vectors representing each person. Each dimension of the vector represented a clinical or lifestyle variable of interest that we considered important. These variables were age, sex, BMI, smoking (pack-years), alcohol consumption, consumption of proton pump inhibitor (PPI) medication and accumulated incidences of heartburn. The dimensions were normalized and then weighted by importance. A participant in the esophageal AdCa group was selected at random, their closest neighbor from each clinical diagnosis group was found and this cluster was then set aside. The process was repeated until the AdCa samples were all matched (Table 1).

**Table 1.** Risk stratification of patients and controls in the discovery, testing and independent validation datasets.

		Discovery		Testing		Validation	
	Weight factor	Cancer (n = 51)	Non-cancer (n = 107)	Cancer (n = 18)	Non-cancer (n = 34)	Cancer (n = 19)	Non-cancer (n = 27)
Diagnoses		IMC: 16 Invasive: 35	HV: 47 NPD: 27 NDBE: 33	IMC: 6 Invasive: 12	HV: 15 NPD: 8 NDBE: 11	IMC: 6 Invasive: 13	HV: 12 NPD: 7 NDBE: 8
Sex: female	5	14	34	4	8	2	9
Sex: male	5	37	73	14	26	17	18
Mean age (years)	3	70.9 (± 8.80)	63.2 (± 13.4)	69.0 (± 10.8)	60.5 (± 12.6)	70.9 (± 7.01)	63.0 (± 9.26)
BMI (kg/m^2^)	3	28.2 (± 4.83)	26.8 (± 4.67)	27.0 (± 4.66)	26.7 (± 4.63)	26.9 (± 4.08)	28.0 (± 3.23)
PPI intake / pill-years	3	1.72 (± 2.73)	1.24 (± 1.83)	1.25 (± 1.72)	1.40 (± 2.20)	1.92 (± 3.35)	1.33 (± 1.44)
Cigarette smoking / pack-years	1	7.43 (± 14.40)	3.42 (± 7.98)	8.22 (± 10.50)	3.37 (± 7.33)	7.90 (± 10.80)	4.20 (± 8.63)
Total alcohol intake / drink-years	0.5	12.07 (± 10)	11.46 (± 9.5)	9.98 (± 10.1)	10.80 (± 9.5)	12.50 (± 11.9)	10.25 (± 7.7)
Heartburn incidence / incidence years	3	1.14 (± 2.3)	1.24 (± 2.3)	1.56 (± 2.6)	1.20 (± 2.21)	1.02 (± 2.7)	1.04 (± 2.1)

Having shown that the groups had similar epigenetic profiles, IMC and invasive cancer were clustered together, as were HVs, patients who had undergone normal endoscopy and had NPD, and patients with NDBE.

All covariates are shown together with the mean values and standard deviation in brackets for each group.

HV: Healthy volunteer; IMC: Intramucosal esophageal adenocarcinoma; NDBE: Nondysplastic Barrett’s esophagus; NPD: No positive diagnosis; PPI: Proton pump inhibitor; SD: Standard deviation.

The second step of array plate design involved assigning all samples to a particular row of the array and adjusting the composition of subjects on the two plates so that their age, sex, smoking, drinking, heartburn and PPI characteristics were as close in value as possible. Matched participant groups were randomly assigned a row on the plate. A pair of randomly selected samples were then swapped and tested. If the swap increased global covariate heterogeneity, the swap was kept. Heterogeneity here was defined through standard statistical tests (*t*-test and χ-square).

### Processing of arrays

The data were analyzed in the R statistical programming environment [28]. Array *.idat* files were loaded into the environment via the *minfi* package [29]. Annotation of arrays was provided by the *IlluminaHumanMethylationEPICanno.ilm10b4.hg19* package [5,30]. QC was performed using the *ShinyQC* package [31], which produces QC measures for the scanning and hybridization stages, as well as a general summative QC measure and various measures associated with control probes. It also provides density plots of the data. An initial round of filtering removed samples that were strong outliers by visual inspection of their M- and β-values when viewed on a density plot. Samples that failed to show a maximum peak around an M-value of -5 while producing sizagingable and anomalous-looking peaks at other values were considered to have systematic errors and these were removed. Further inspection of individual QC measures caused more arrays to be removed if they were obvious outliers in more than one measure; however, particular importance was attached to the bisulfite conversion measures, and all outliers on this measure were removed. Specifically, any bisulfite conversion with control intensities lower than 2000 were automatically removed even if other QC measures indicated no issues. In total, 109 samples were removed for QC reasons, leaving 275 samples for final analysis.

The data were normalized using the functional normalization method implemented in the R *minfi* package. This method was tested and recommended by Fortin *et al.* [6,32] as appropriate for datasets with global methylation changes, such as those encountered in cancer-related data, and thereby deemed the most appropriate method. Sample cell-type heterogeneity can cause patterns of methylation unrelated to clinical diagnosis phenotype and thereby increase type I errors if it is not addressed. The *EpiDish* R package [7,8,33,34] estimates cell type fractions from the data using key indicator probes. Epithelial cell type fraction was the main variable used from this result. The quantities of fibroblast cells were always very small (mean content value = 0.005), meaning that the immune cell (IC) type composition percentage was effectively an inverse mirror of epithelial cell content. If epithelial cell content was adjusted for in the analysis, IC cell content would also be adjusted. Additionally, we calculated the epigenetic ‘Horvath age’ values from the data and plotted them against the reported age values of the participants [35].

### Batch effect adjustment

**Figure 3.**
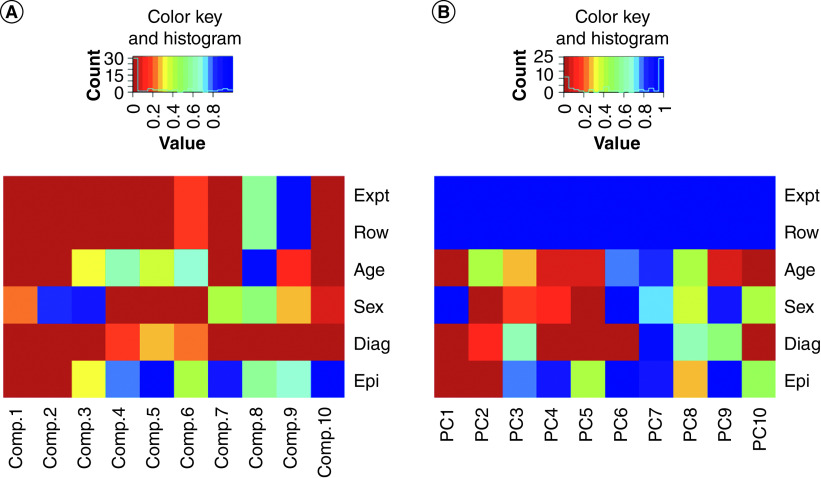
Heat map plot of p-values of first ten principal component analysis components (A) before and (B) after plate-fitted residual correction for experiment batch, array row, age, sex, disease diagnosis and epithelial cell content. The components were statistically tested against experiment, array plate row, age, sex, disease status and epithelial cell content. The plate-fitted residuals show p = 1.00. Blue = low p-value; red = high p-value.

We observed strong batch effects in the normalized data. Singular value decomposition (SVD) of the data showed that experimental batch variables, namely the array rows and the plates, were both strongly significantly associated with higher order SVD components (second and third components). We attempted to remove the effects of these with the R *ComBat* package [36]. However, calculation of SVD components after *ComBat* had been applied still showed strong batch effects and we considered that a more stringent method of batch removal was necessary. A linear model based on the array rows was fitted to every probe on the array and the residuals were taken. There was no detectable presence of any batch variable after this had been performed (none of the 20 highest SVD components had any batch-associated statistical significance). Encouragingly, the highest-order SVD component of the batch-fitted residuals was now significant with the clinical diagnosis group (p < 1 × 10^-4^). All subsequent calculations worked with the residuals of a batch-fitted linear model. Figure 3A & B show heat map plots of the batch variables before and after complete removal of both row batch and experiment batch; the higher-order components retain strong significance with the disease and other covariates after this adjustment.

### Weighted gene coexpression network analysis

We performed the main analysis using weighted gene coexpression network analysis (WGCNA). This technique was originally developed for the analysis of gene expression data but has seen more recent application in methylation data [37]. The aim of the technique is to find modules of genes that are expressed in a similar manner across experimental replicates. Although methylation arrays survey both coding and noncoding regions, we reduced the analysis dataset to the gene level as a first point of analysis. There are practical and theoretical reasons why this was done. Firstly, the analysis of methylation networks is in a state of relative infancy compared with that of gene networks, and their relevance and importance are not as well established because of this. We would therefore have more confidence in the validity of any conclusions and their connection to the disease if we worked at the gene level. Secondly, and perhaps most importantly, there are genuine concerns about the reproducibility of methylation arrays in general [9,38], so by collapsing loci to the level of the gene and selecting the locus with the most stable behavior, the chances of reproducibility were greatly increased.

### Assessing robustness of the data

As well as the technical replication issues mentioned above, another issue that is potentially problematic for any analysis of samples drawn from a subpopulation is the question of how robust the data are to small changes in cohort. This is, arguably, an issue that does not receive enough attention, yet many published analyses fail to show reproducibility in the wider population [39]. It is obviously not possible to say in advance whether an analysis is robust in the outside world, but some assessment of the *internal* robustness of an analysis can be performed by considering what happens if small changes are made to the composition of the input data. If certain genes or gene modules, for example, disappear when the analysis is repeated after the removal of a very small number of samples, this is likely to be an indication that the analysis was too finely fitted to the particular cohort being analyzed. We therefore modified the standard WGCNA pipeline to address the issue of robustness by repeating the analysis several hundred times with minor modifications to the discovery dataset, and used clustering methods to summarize the results.

### Mapping methylation data to single gene values

Annotation for the methylation locus was provided by the same R package used for QC: *IlluminaHumanMethylationEPICanno.ilm10b4.hg19*. We selected methylation loci that had a GenBank mapping, and all other probes were discarded. This meant that the 169,185 methylation loci had been isolated to 13,910 genes. Usually, several probes map to a single gene, and we reduced the data further by using the CollapseRows function on the WGCNA R package, which chooses a representative probe for each gene [40]. This is a necessary procedure for an analysis in WGCNA as genes cannot have multiple module membership. The selected methodology for this selection is MaxMean method (the function default), which goes through all the probes of a single gene and selects the one with the highest mean among all samples.

Selection of the WGCNA parameters, specifically the scale independence value, was performed using the entire dataset. The WGCNA methodology raises correlation coefficients to a power in order to demarcate module boundaries more clearly and to bring the network closer to scale-free topology. It is necessary to determine in advance a suitable power. This was performed and a value of 6 was selected. This value was maintained through repeated WGCNA calculations. We therefore made the assumption that the removal of five individuals in a cohort of over 100 individuals would not radically alter the network topology. Every constructed network had a scale factor of 6.

A succession of networks was constructed by removing just five randomly selected samples at a time. The number of permutations possible is such that the probability of removing the same five samples when performed a few hundred times is almost zero. We constructed the WGCNA modules and determined module membership of all the genes and noted this (it was the basis of the module clustering performed later on). The module Eigengene was determined, and each module was statistically tested for significance via logistic regression against the Eigengene. The logistic regression model was fitted to clinical diagnosis but also to age, sex and epithelial cell content. This meant that a module had to be significant for the disease once these covariates had been accounted for.

### Determination of important genes in modules

We took the results of the module adjacency function and normalized it to an upper bound of one to make it independent of module size and took the reciprocal so that the hub gene had a value of 1 and less significant genes were larger. We then multiplied this normalized adjacency gene value by the p-value of the entire module which was generated by the logistic regression testing of the module Eigengene. The hub gene would retain the p-value of the module, but the modified adjacency function would inflate the values of other genes. This is no longer a p-value, but is instead a very useful numerical indicator of whether a gene belongs to a significant network and whether its role in that network is important. This metric value was noted for every single gene, across every instance of network construction. Those genes that failed to gain an assignment to a module were given a default value of 200, which was similar to the metric value of the least important gene in the least significant network. Averages were then taken. If a gene was routinely scoring low values of the modified p-value metric across hundreds of instances of classification, we considered this to be an important gene that is robustly associated with disease.

### Clustering of modules

**Figure 4.**
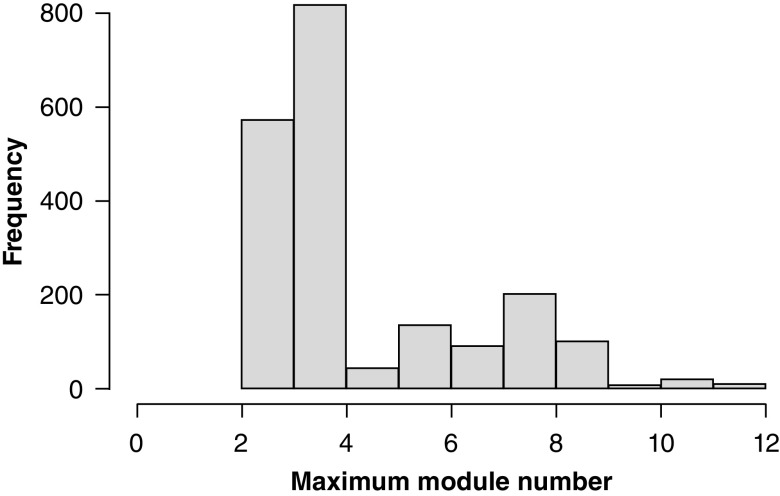
Module membership after repeated classification with modification of five samples (3%) of the discovery cohort.

Selecting the best genes from each module after creating hundreds of networks is less straightforward because the number of modules in each network is not constant. Figure 4 shows a histogram of the distributions of the module membership for the 5000 most significant genes after all rounds of WGCNA module creation. What was unexpected was the extent of the variation; a very small change in cohort composition of just five samples (3% of the entire discovery cohort) can make a WGCNA change from creating two modules to creating 12 using the exact same parameters. In each occasion of module generation, the modules are created and numbered in order of size, with some genes often appearing together in the same module, but that module is numbered differently at different instances of WGCNA module creation. These changes and similarities can be tracked by performing k-means clustering based on the genes’ module assignments over all rounds of network construction. If a group of genes is consistently forming a meaningful module, but that module is assigned a different identity, clustering will retain the broader composition of that module.

### Generation of candidate genes from the modules

We decided to rank the modules based on the best (i.e., lowest) scores of the genes in the module. Module clusters with the lowest-scoring genes are assumed to be the best modules, which means that module clusters can be ranked. The best genes from every module cluster could have been selected, but from module testing we know that some modules repeatedly show no significance with disease. We implemented a simple selection method where we selected N genes (where N is a predetermined number) from the best module and (N − 1) genes from the second-best module and continued until N = 1. We therefore have several ways of creating gene lists based on the k-value of the clustering and the initial N value describing the number of genes selected from the most significant module. We investigated all possible classifiers from k = 2 to 20 and N = 2 to k.

### Selecting the best gene list

Testing of the data was performed using the R-Caret package [10,41] implementing a random forest classifier. Candidate probes generated a classification model using the discovery dataset and also the covariate information (age, sex and epithelial cell content). Receiver operating characteristic (ROC) curves and area under the curve (AUC) values were determined for all three datasets. For the training data, a tenfold cross-validation model was produced, while for the test and independent validation datasets the data were assessed using the model established from the discovery data.

By proposing a relatively large number of candidate gene lists, there is a risk that the best-performing classifier could be specific to the dataset in question. We therefore calculated the average AUC of all three classifiers and also the variance of the average of all three classifiers and added these ranks together and selected the classifier with the lowest sum. This means that we are choosing a classifier that works well but that also works consistently across the three datasets, making the discovery of the classifier less likely to be a chance discovery.

Given the relatively high values for the ROC, it is possible to bias the model toward finding cases of higher interest, such as cancer. We therefore added a wrapper cost model to bias the classification [42]. We aimed for a minimum sensitivity to detect cancer of 0.9. We found that biasing cancer cases as being 20-times more important than non-cancer (i.e., a weight of 20) ensured this threshold. Adding a wrapper to a model can change its overall accuracy, and we took care to minimize such negative effects by manually setting the weights.

## Results

### Participants

The cohort-wide male-to-female ratio was 2.52, which reflects the male preponderance of the disease; every discovery, testing and validation group in our cohort had more male subjects, in both controls and cancer groups. Participants completed a questionnaire prior to sample collection which included the recording of age and sex information. There were no missing data on age or sex.

### Horvath age calculation

We calculated the epigenetic Horvath age values [11,43] from the data and plotted them against the reported age values of the participants. We observed no significant deviations between them (Supplementary Figure 1).

### Assessing reproducibility in the data using technical replicates

We tested ten technical replicate samples on different array plates, which were collected at different times. We calculated the mean absolute difference in β-values across all probes that had passed QC and compared them with their corresponding replicates. Only four of the ten duplicate pairs replicated well (mean absolute difference <0.01). We also calculated a composite array for data comparison purposes, which was derived from the mean absolute difference calculated across all other samples in the experiment. There was a tendency for arrays that had a mean absolute difference that was greater than that of the composite array to also replicate badly. We did observe that the pairs that replicated well had an average β-KS distance of between 0.3 and 0.6 and that the worst-performing pairs contained single samples that exceeded 0.6. We could not use duplicate pairs in the study and needed to select an individual one. We therefore used the array that had the lower KS distance of the duplicate pairs. We did not apply the discarding of arrays based on the KS distance in general.

### Creation of classification outcome groups

There were three classes of non-disease control (74 HVs, 42 subjects with no positive diagnosis of abnormalities after endoscopy and 52 samples from people with nondysplastic Barrett’s esophagus [NDBE]). There were 88 individuals in the cancer group, which comprised 28 people with IMC and 60 with invasive esophageal AdCa, which was defined as disease extending to the submucosa or more extensively [9]. All the cancer patients were newly diagnosed and none had yet received any cancer therapy. The nine HGD samples represented a potential categorical ambiguity; we therefore decided to omit them during the discovery stage. This agrees with the clinical finding that approximately 40% of patients with HGD in Barrett’s esophagus progress to AdCa within 5 years [4].

### Creation of analysis cohorts

The entire cohort was divided into three subgroups: one large group for classifier discovery, a testing group and a validation group. The discovery cohort comprised 158 people (64% of total available participant data), and the two testing and validation cohorts contained 52 and 46 people, respectively (20% and 16% of participant data). Patient samples were stratified by disease status then selected randomly, to ensure similar ratios in all the training and testing groups [41].

Table 1 show the mean ages of cancer and non-cancer subjects, as well as the mean values for other factors used in matching (BMI, PPI intake, cigarette smoking, alcohol consumption and heartburn incidence). These latter variables represent accumulated measures. The pack year measure for accumulated smoking is a commonly measure of cigarette consumption [44]. We produced analogous measures for alcohol consumption (drink years), PPI intake (pill years) and heartburn (incidence years) based on frequency and duration information reported by individuals.

We aimed to match participants in cancer and non-cancer groups by a combination of all the factors. This was considered important as although there was a broad agreement of reported age with epigenetic age (see Supplementary Figure 1), there is still some variation. It is known that lifestyle factors such as cigarette and alcohol consumption can accelerate epigenetic aging (and have their own unique effects that need to be matched [14,15,45,46]). We have therefore taken all these factors into account in producing a matched pairs so that even though an older cancer subject at times may be matched with a younger person if (for example) their combination of lifestyle factors and of reported symptoms and medication (heartburn, PPI intake) would potentially make them more epigenetically similar. When the broader situation of lifestyle, symptoms and medication is viewed, we found that the variation in our broader cohort is generally modest. To assess this, we selected random pairs of individuals in the cohort to see how “badly” they would mismatch, using the distance-based matching used to match subjects. Using 100,000 randomly selected pairs of individuals. We found an average mismatch distance of 2.95. This compares to the average mismatch distance of our pairs of 0.352.

Cancer subjects were very carefully matched by all variables, including age, with three subclasses of control (HV, no positive diagnosis and nondysplastic Barrett’s esophagus). During matching, AdCa and IMC individuals were matched together against the three subclasses of control, with HGD matched last. Most of the individuals in the AdCa and IMC groups of five had an age range of less than 10 years from each other. There are, however, additional controls who have tended to be younger on average, as can be observed in the means of ages in Table 1. These additional younger people in the controls had originally been matched against the HGD group, which was younger on average than AdCa and IMC. There were also fewer available older controls to match against them as AdCa and IMC subjects had been matched first.

We did not remove these additional younger controls because it would represent a substantial loss of statistical power. These additional younger people have tended to possess above-average values of BMI and of cigarette, alcohol and PPI consumption as well as greater incidences of reported heartburn for their age, as this reflects the values in the HGD group they were matched against. It is likely, therefore, that genetic ageing effects have been accelerated in these younger people, which will offer some mitigation against the age differences between cancer and non-cancer groups; however, we recognize that it is imperative that any subsequent fitted statistical models should include and adjust for age as a covariate so that probe values are unable to use it as a proxy for disease.

During initial data processing, we investigated methods for removing the batch effects of the arrays, which from principal component analysis were found to be strong, but found that many conventional methods of correction still left observable batch effects in the data (Figure 3). Taking residuals from a batch-fitted linear model, based on each row of the array, removed any detectable traces of batch effect (confirmed with principal component analysis), both within the array plate and between all the plates. All subsequent analyses were performed on batch-corrected data produced in this manner.

Methylation probes were mapped to genes, and a representative gene probe was used based on the probe with the largest mean across all samples. WGCNA was performed repeatedly on the discovery cohort with slight variations in cohort each time, achieved by removing five randomly selected samples (3% of the cohort) and repeating 2500 times. This was performed to discover those probes that were repeatedly being shown as significant despite this small variation, thereby reducing the possibility of type I errors. WGCNA generates modules of genes. Typically, between two and three modules were generated, with a maximum instance of 12 modules created. The histogram of the distribution of all maximum module values generated is shown in Figure 4.

WGCNA module membership was noted for every gene over every instance of WGCNA. In order to determine which genes were repeatedly being assigned to the same module, the WGCNA module membership information was clustered using k-means clustering. This is necessary because the WGCNA module numbering for individual genes might change in response to small variations in the cohort. There are several reasons for this: larger modules might sometimes be broken apart, for example, or some genes might not qualify for any module on occasion. The clustering is able to identify any groups of genes that were exhibiting similar module assignment behavior together. It is also possible to see any instances where larger modules were broken up by adjusting the parameters of the clustering.

**Figure 5.**
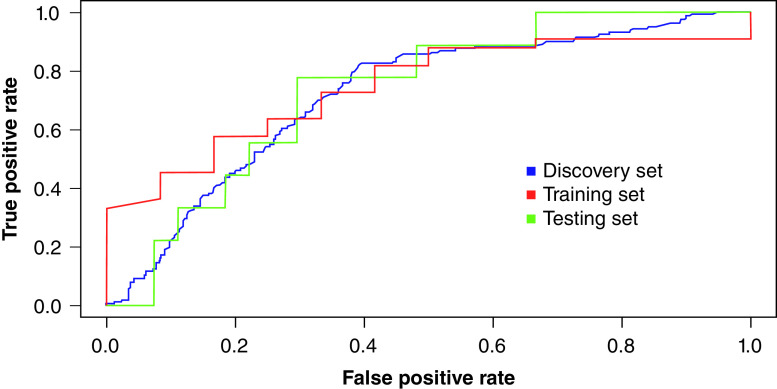
Receiver operating characteristic curve of the most successful classifiers for the discovery set, testing set and validation dataset.

The classifier models included all the covariates used for original matching of patients with controls. We had expected that they would disappear from the final algorithm but this was not the case. Age and sex remained important independent features. The performance of all classifiers was ranked according to the mean AUC of the three classifiers and by variance between the training, testing and validation datasets. We therefore defined the most successful classifier as the one with the best combination of high-ranking mean and low-ranking variance. The AUC was used as the classifier performance metric, and the results of the single best-performing classifier are displayed in Table 2, although multiple models with parameters different from the defaults yield similar results. For example, we also created random forest prediction models with values for the *mtry* and *ntree* parameters within Caret other than the default, but noted no significant differences. The ROC curve is shown in Figure 5. For the training set, the diagnostic accuracy was 0.73 (95% CI: 0.69–0.75), with an AUC also of 0.73 (95% CI: 0.69–0.75). The findings were very similar in the testing set, with an accuracy of 0.69 (95% CI: 0.66–0.72) and AUC of 0.72 (95% CI: 0.68–0.74), and the validation set, with an accuracy of 0.65 (95% CI: 0.61–0.70) and AUC of 0.73 (95% CI: 0.68–0.75). Using the base model, the training set had a specificity of 0.94, and this increased to 0.97 in the testing and independent validation sets. To test the effectiveness of our models to find all cases of cancer, we considered a cost of 20-times more for cancer instances than non-cancer ones using the cost-sensitive wrapper algorithm. This yielded a sensitivity of 1.0 within the discovery dataset and values of 0.94 and 0.91 within the test and validation datasets, respectively, and the specificity dropped to between 0.10 and 0.31. Note that the ROC of these results is slightly less than the nonweighted versions as the wrapper algorithm used to generate these results is biased for finding the cancer cases. There was no difference in discriminatory ability of the epigenetic panel between cancer versus HVs or cancer versus benign disease.

**Table 2.** Area under the curve for training set and the two holdout sets.

	Base model: IMC and invasive cancer combined			Base model: IMC only	Base model: invasive AdCa only	Cost-sensitive model: IMC and invasive AdCa combined			Cost-sensitive model: IMC only	Cost-sensitive model: invasive AdCa only
	AUC	Sensitivity	Specificity	Sensitivity	Sensitivity	AUC	Sensitivity	Specificity	Sensitivity	Sensitivity
Training	0.73 (0.69–0.75)	0.28 (0.26–0.30)	0.94 (0.91–0.98)	0.55 (0.51–0.59)	0.18 (0.15–0.21)	0.72 (0.69–0.76)	1.00 (0.98–1.00)	0.10 (0.06–0.16)	1.00 (0.96–1.00)	1.00 (0.97–1.00)
Test	0.72 (0.68–0.74)	0.11 (0.09–0.15)	0.97 (0.94–1.00)	0.25 (0.21–0.29)	0.00 (0.00–0.03)	0.72 (0.68–0.75)	0.88 (0.84–0.91)	0.31 (0.26–0.36)	0.78 (0.74–0.82)	1.00 (0.96–1.00)
Validation	0.73 (0.68–0.75)	0.19 (0.16–0.23)	0.97 (0.04–1.00)	0.29 (0.25–0.32)	0.14 (0.11–0.18)	0.63 (0.59–0.68)	0.86 (0.82–0.90)	0.23 (0.18–0.29)	0.86 (0.82–0.91)	0.86 (0.81–0.89)

All results are mean (95% CI).

Showing tenfold cross validation for the base model and after a cost-sensitive wrapper algorithm is applied which generates a high cost for missing cancer cases.

AdCa: Adenocarcinoma; AUC: Area under the curve; IMC: Intramucosal esophageal adenocarcinoma.

To assess the individual effects of the methylation data and the covariate data, we performed separate classification using only probes and only the covariates (Table 3). In each instance, classification was above the level of random assignment. We also investigated using probe and covariate information but without epithelial cell content. ROC curves for these different analyses are shown in Supplementary Figure 2. In all cases, the epigenetic probes performed better than the covariates alone, as expected, but the combination performed better still (Table 3).

**Table 3.** Area under the curve using only probes and only covariates of age and sex.

	Probes only	Covariates only
	AUC	AUC
Discovery	0.65 (0.60–0.69)	0.63 (0.59–0.68)
Testing	0.64 (0.60–0.70)	0.61 (0.56–0.66)
Validation	0.64 (0.59–0.70)	0.60 (0.54–0.65)

All results are mean (95% CI).

Table for training set (tenfold cross validation) and the two holdout sets.

AUC: Area under the curve.

### Intramucosal & AdCa samples

We looked at the classification results for a range of the best-performing classifiers using the base and cost-sensitive models derived from the discovery dataset (Table 2). In the base model, sensitivity was very low but better for IMC (0.29–0.55) compared with the invasive cancer group (0.00–0.18). In the cost-sensitive model, sensitivity was very high for both IMC (0.78–1.00) and invasive cancer (0.86–1.00).

### HGD samples

Classification of the nine HGD samples was performed using several competing candidate classifiers from the discovery dataset with a random forest classifier. Using the base model, none of the HGD samples was classified as cancer. Once again, as we raised the cost of missing cancer cases, the ability to find HGD cases changed. At a cost set at 20, the cost-based model found 100% of the HGD cases as well. The progressions between these two cost extremes are shown in Supplementary Table 1.

## Discussion

This is the first paper to demonstrate the possibility of a clinically viable tool to detect patients with esophageal AdCa, based purely on the epigenetic profile contained in a saliva sample together with age and sex information. In three independent datasets which were matched for patient characteristics, an algorithm was generated, tested and validated and had an overall accuracy for identifying esophageal AdCa of 69–72% based on the ROC. Using a cost-sensitive wrapper algorithm, it is possible to detect 86–100% of all cancer cases if aimed at a symptomatic patient group where sensitivity is the key variable. It may also be possible to use this same test for screening, as the base model offers a specificity of 94–97%, with a sensitivity for detecting disease of 11–28%. These findings are very similar to those used in the UK bowel cancer screening program [47]. Some of the seven probes that we found have been individually linked to other cancers, but ontology analysis of the gene panel showed that these seven loci do not offer any statistical evidence of being associated with any other cancers. We have not specified the specific probes here because, while the ones chosen are representative of particular genes, others could be substituted which would work almost as well. Furthermore, the small number does not permit any meaningful insight into the etiopathogenesis of the cancer itself.

Our saliva-based epigenetic algorithm works particularly well in patients with early disease and in those with premalignant changes of HGD, where early intervention can dramatically reduce the risk of dying [48]. Using a cost-sensitive model, the classification of both IMC and HGD samples was 78–100%. This indicates that early disease is almost as successfully detected as cancer cases. We plan to further study this point in the future. It is also very important as the crucial cases to detect are the early cancers that are potentially curable. No reliable noninvasive test currently exists for these important groups.

The case–control matching combined a variety of covariates, which led to a certain disparity of age between case and control groups. This meant that, for example, two people of different ages were matched if the elder case sample were to be matched with someone with less healthy characteristics in the control group (smoking, alcohol consumption). In further studies, it seems reasonable to match subjects for similar lifestyle properties, but perhaps age matching of subjects should be given a higher priority.

A practical diagnostic tool would involve a fully trained classifier such as the random forest classifier that was used based on the discovery loci. In theory, batch correction would not be necessary, as it is a single measurement. As we used the fitted residuals of the batch variable, this means that average values of the whole batch-adjusted array were effectively zero and a baseline adjustment would therefore need to be applied.

Diagnostic and prognostic success has been achieved using tissue sample methylation. Li *et al.* were able to produce a classifier that could distinguish between Barrett’s esophagus, AdCa samples and squamous cell carcinoma from normal tissue with a high level of success (AUC = 0.992) [16,49]. Biomarkers for esophageal cancer have been discovered both in the tissue of origin and in blood. Salta *et al.* discovered individual biomarkers in tissue with the best-performing of them (ZNF569) able to differentiate cancer samples from non-cancer samples with an accuracy of 79% (AUC = 0.85) [50]. Qin *et al.* found a panel of five markers present in blood plasma with an AUC of 0.93 [17,51]. Both these studies did combine AdCa and squamous-cell types in their cases, which have considerable histological and biomolecular differences from each other [52].

Methylation-based biomarkers in saliva specifically have found comparable levels of accuracy in discriminating samples of other cancers from controls. For example, Lim *et al.* evaluated a panel of five tumor-suppressor genes isolated from saliva to discriminate human head and neck cancers from controls for a cohort of human papillomavirus (HPV)-negative and -positive individuals (AUC for HPV-negative = 0.86 and HPV-positive = 0.80) [3,53].

In our study, the WGCNA methodology has demonstrated its usefulness in determining a set of candidate probes very efficiently. It appears to have certain inherent advantages over generating candidate probes from a two-group analysis. First, the collapsing of data to a single representative probe has meant that an individual probe has had to demonstrate a certain reliability against what are effectively competing pseudo-replicates. Second, the distinctness of the modules means that probes from each module are also exhibiting more distinct behavior. Often, the genes at the top of a two-group analysis are probes from the same entity (gene or CpG island). This is desirable to bolster evidence, but for diagnostic purposes, if the probe is reliable, there is no need to replicate it. What would be desirable is to survey all the possible ranges of inherent biological variation to ascertain disease status. WGCNA, with its distinct modules, is arguably in a better position to do this. It could be possible to ascertain more biologically variant probes in a two-group gene list, but such post-processing procedures would be equivalent to what WGCNA is doing in the first place.

The covariate data had limited predictive value, due perhaps to the age and sex matching. In a clinical setting, age and sex information will be freely available.

This initial analysis has been restricted to the gene level, but further scope for this study could be achieved using the level of methylation loci instead. It would, however, require special consideration of not only the much increased demands in computational facilities but also the vast increases in variables which could make the scope for erroneous (i.e., type I) discoveries considerably greater.

### Limitations of the study

These promising results suggest that information relating to esophageal cancer status can be ascertained from a saliva sample. This study is an initial one that has deliberately focused on a set of probes associated with specific genes collapsed to a single representative probe. For now, we have disregarded the rest of the data due to concerns about reproducibility, but it could be possible to make further improvements to the study by incorporating the considerable amount of data present in these other regions. This would be a considerably larger computational task to implement, and the manifold increase in candidate probes could also have repercussions in the generation of an increased number of type I errors, although these would be mitigated by including a larger training dataset and more independent validation datasets.

The weighting of all the different covariates for case–control matching has meant that certain factors, such as age, were not as closely matched as they could be but were instead matched based on certain lifestyle similarities. Whether so many covariates should be taken into account is, perhaps, overambitious given the limitations of the available subjects in the study. It might be better in follow-up studies to more closely match factors such as age.

Array-based studies (and all studies that survey the entire genome or methylome) are at great risk of statistical errors due to the number of variables being far in excess of the number of replicates. We have worked to reduce this by operating at the level of the gene. In any investigative study of this nature, with limited space for sample interrogation, it is necessary to greatly increase the number of disease cases in the discovery set to a ratio that is significantly higher than in the population. Based on the performance of our test, it is likely to be used as a diagnostic triage tool for symptomatic patients, and in such instances the number of disease cases and people with esophageal abnormalities will also be inflated.

We do not yet know whether the test is specific for esophageal AdCa, although, given the number of possible classifiers we have generated, this is likely to be the case for at least some of them. Furthermore, an array-based test is not currently viable in a clinical setting as it is too expensive. However, it should be possible to dramatically reduce costs if the test can be reliably reproduced using the small number of epigenetic markers with a cheaper technology such as amplicon-based sequencing or other newer high-throughput testing platforms that are becoming available.

### Diagnostic implications

The development of diagnostic triaging tests based on saliva could produce substantial benefits to healthcare providers and patients. Most of the methods currently required for the accurate diagnosis of esophageal cancer involve procedures that are invasive, uncomfortable, anxiety-inducing, time-consuming, costly and limited by the availability of trained personnel. This in turn has the potential to make such procedures less likely to be recommended by the clinician, or agreed to by the patient, unless symptoms are particularly self-evident and persistent [18,54]. This creates a situation where those individuals whose symptoms are advanced are prioritized for diagnosis. This is understandable, but it happens at the expense of individuals who might accrue substantial, and maybe even greater, benefits from a diagnostic appointment when the disease is at an earlier stage. A simple diagnostic triaging test would therefore be valuable. The cytosponge is currently being examined in this role, but it too is invasive as patients have to swallow a capsule on a string and then have it removed [55,56]. Our simple saliva test could be used to triage patients at risk for cancer who are being considered for endoscopy. Although this test has an accuracy of only around 72%, the cutoff can be set to achieve a sensitivity of 98%, which would currently give a specificity of 10%. If sensitivity were lowered to 90%, specificity would reach more than 30%, and the test has not yet been optimized. These results could lead to a 10–30% reduction in endoscopies performed in patients referred with suspected esophageal cancer. In the UK alone, 1 million endoscopies are undertaken annually, and around 50% of these are to exclude cancer [57]. Worldwide, the impact of a simple saliva-based screening test would therefore be enormous, particularly as the situation – where diagnostics are only implemented when it is too late to help – will only get worse in the short term as backlogs in many healthcare systems are now reaching unprecedented levels [57–59].

Another issue that saliva-based diagnostic tests address which is arguably not given enough consideration at present is that saliva collection does not require needles. Surveys have indicated that around 10% of the population have a significant fear of them [19,60] and that this fear actually causes people to avoid preventative care [61].

Any rapid, noninvasive, easy-to-administer test that can provide intelligent prioritization of limited human and material healthcare resources therefore has the potential to save lives. Saliva-based epigenetics have shown exciting potential, and we consider that bringing this technology to a clinical diagnostic setting would not just be useful but is actually an urgent necessity to alleviate some very pressing problems.

## Conclusion

This study has demonstrated the potential for saliva as a diagnostic material whose potential is just starting to be explored. Using gene-based methylation information, we have shown that there is information about disease status in the methylome. Further incorporation of other methylation data that were not used in the study has the potential to improve prediction accuracy, but due attention will need to be paid to reproducibility.

**Summary points**

- Saliva-based tests are noninvasive and easy for patients to do at home.
- Saliva-based cancer biomarker panels have been developed for head and neck cancers.
- We aimed to develop a saliva-based epigenetic panel to identify patients with esophageal adenocarcinoma.
- We collected samples from 88 patients and 168 controls.
- We used Illumina EPIC methylation arrays, with 850,000 individual methylation loci.
- Stringent quality control measures were used.
- We found a seven-probe epigenetic panel which had a diagnostic accuracy of 73% in a discovery cohort of patients.
- This panel was replicated in a testing cohort and an independent validation cohort.
