# Supplementary figures and images for: Using saliva epigenetic data to develop and validate a multivariable predictor of esophageal cancer status
Source: Epigenomics. 2024 Jan 16;16(2):109–25. doi: 10.2217/epi-2023-0248 (PMC10825730; doi:10.2217/epi-2023-0248)

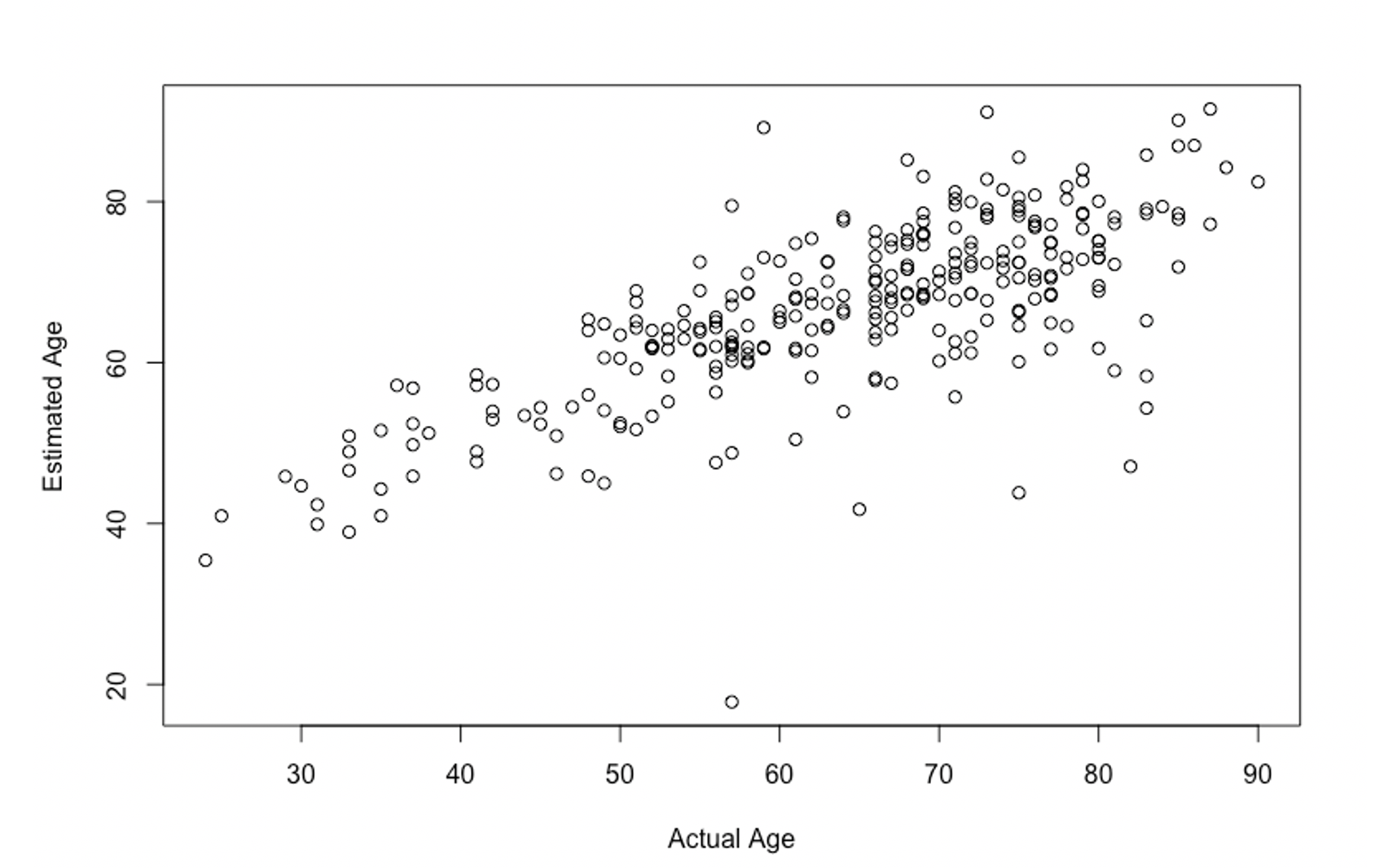

Supplement: Supplementary file 1 [file epi-16-109-s1.png]

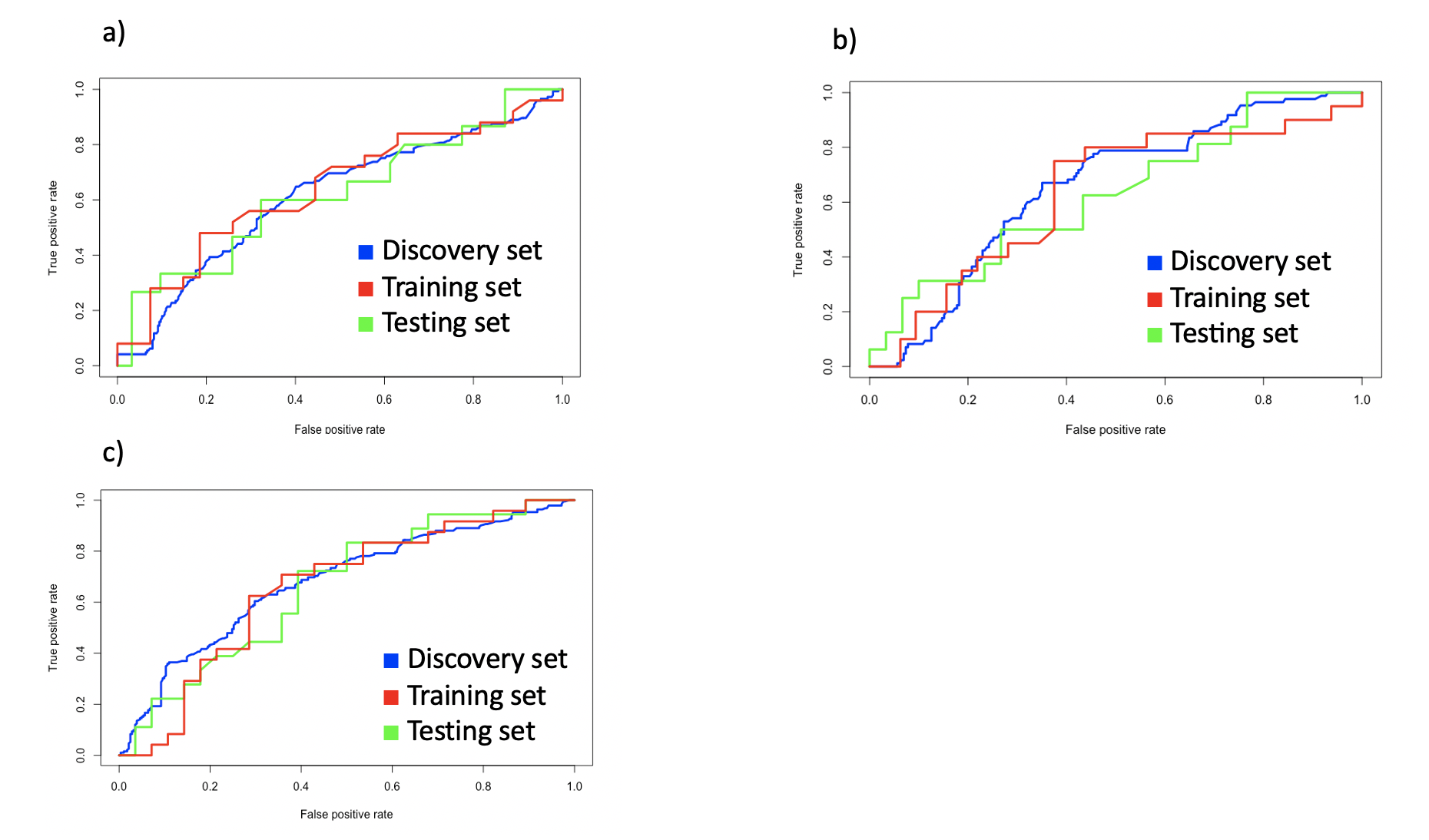

Supplement: Supplementary file 2 [file epi-16-109-s2.png]
