# Supplementary material for: Using saliva epigenetic data to develop and validate a multivariable predictor of esophageal cancer status
Source: Epigenomics. 2024 Jan 16;16(2):109–25. doi: 10.2217/epi-2023-0248 (PMC10825730; doi:10.2217/epi-2023-0248)
Supplement: Supplementary file 3 [file epi-16-109-s3.docx]

***Supplementary Table 1: Classification results for high-grade dysplasia cases:***

| Weight | 0 | 5 | 10 | 15 | 20 |
| --- | --- | --- | --- | --- | --- |
| Classified as Cancer | 0% | 67% | 80% | 100% | 100% |

*High-grade dysplasia cases arising in Barrett’s oesophagus are an intermediate group with a 40% risk of developing cancer at 5 years. The data we held on 9 HGD patients was withheld from the training algorithm. When used for testing, the likelihood of detecting these patients rose together with the weight applied to the cost-sensitive wrapper algorithm in line with the likelihood of detecting cancer.*
